# Comparison of tip-bendable aspiration-assisted and standard access sheaths in the treatment of lower calyceal stones

**DOI:** 10.1590/1806-9282.20241033

**Published:** 2024-12-16

**Authors:** Mehmet Uslu, Ümit Yildirim, Mehmet Ezer, Ömer Erkam Arslan, Hikmet Yaşar, Kemal Sarica

**Affiliations:** 1 Kafkas University, Medical School, Department of Urology – Kars, Turkey.; 2 Antalya Kepez State Hospital, Department of Urology – Antalya, Turkey.; 3 Sancaktepe Şehit Prof. Dr. Ilhan Varank Training and Research Hospital, Department of Urology – İstanbul, Turkey.; 4 Biruni University, Medical School, Department of Urology – İstanbul, Turkey.

**Keywords:** Ureteroscopy, Calculi, Renal, Laser lithotripsy

## Abstract

**OBJECTIVE:**

The aim of this study was to compare the success and complication rates of retrograde intrarenal surgery for lower calyceal renal stones performed with novel tip-bendable suction ureteral access sheaths and standard ureteral access sheaths.

**METHODS:**

Between March 2023 and March 2024, a total of 88 patients underwent retrograde intrarenal surgery for lower renal calyceal stones smaller than 20 mm. Based on the access sheath used, patients were divided into two groups: Group 1 (n=43) included patients treated with novel tip-bendable suction ureteral access sheaths and Group 2 (n=45) included patients treated with standard ureteral access sheaths. The pre- and postoperative data of the two groups were compared. Complications were assessed by using the Modified Clavine Dindo system, and stone-free rate was assessed after 4 weeks by using non-contrast computed tomography.

**RESULTS:**

There was no significant difference between the groups regarding demographic data or preoperative laboratory results, and the median stone size was comparable in both groups. The stone-free rate did not show any significant difference between the two groups. However, the median residual stone size was substantially higher in the standard ureteral access sheaths group [4.25- (3.75–5) vs. 6- (5–7), p=0.01] and the surgical duration was less in the novel tip-bendable suction ureteral access sheaths group (p=0.032).

**CONCLUSION:**

Our current findings demonstrate that the use of a new tip-bendable suction-assisted access sheath during retrograde intrarenal surgery in the management of lower calyceal stones less than 2 cm may shorten the operative duration, limit the rate of complications, and end up with smaller residual stone fragments when compared with the use of standard ureteral access sheath.

## INTRODUCTION

Lower calyceal stones constitute approximately 35% of kidney stones, and interventional treatment of these stones has always been a controversial topic for endourology due to the asymptomatic nature in the majority of the cases^1^. Surgical planning involves consideration of certain criteria such as stone size, presence of pain, infection, and obstruction. Accumulated experience so far demonstrated that treatment of such stones may be complicated due to the difficulties in stone removal and less likelihood of fragment elimination as well as anatomical access to the lower part of the renal collecting system^2,3^. Clinical application of retrograde intrarenal surgery (RIRS) has prominently increased in the last two decades due to the significant advancements in instrument technology and the effective use of the holmium-YAG laser for stone disintegration^4^. According to the European Urology Association's Urolithiasis Guidelines, percutaneous nephrolithotomy is the preferred initial treatment for kidney stones larger than 2 cm, whereas extracorporeal shock wave lithotripsy and RIRS are advised as equally effective options for medium-sized stones.

The use of a ureteral access sheath (UAS) during RIRS procedures has the potential to lower intrarenal pressure, enhance endoscopic image quality, and reduce infectious complications following surgery^5^. Many studies have further shown that ureteral access sheaths with aspiration ability are superior to regular ones in several aspects, including reduced operating times, increased stone-free rates, and decreased infectious complications^6–8^. Related to this issue, as a further development in sheath design, recently introduced tip-bendable suction UASs can be specifically directed to the stone-bearing targeted calyx^5^. Despite certain proposed advantages, the potential benefits of this new form of access sheath in the RIRS treatment of lower calyceal stones are not well documented so far, although access is challenging and spontaneous fragment removal is low, as previously emphasized.

In this study, we aimed to compare two different types of access sheaths: the novel tip-bendable suction-assisted ureteral access sheath (NTBS) versus the standard ureteral access sheath (SAS) regarding their possible impact on the success and complications of the RIRS procedure, specifically in the management of lower calyceal stones.

## METHODS

Between March 2023 and March 2024, the medical records of patients undergoing RIRS for isolated lower renal calyceal stones (<20 mm) were recorded prospectively at two specified centers (Kafkas University School of Medicine, Sancaktepe Şehit Prof. Dr. İlhan Varank Training and Research Hospital). All ethical guidelines outlined in the Declaration of Helsinki were adhered to, and the study protocol was approved by the institute's ethics committee (80576354-050-99/415). Patients undergoing RIRS for stones smaller than 20 mm and isolated in the lower calyx met the inclusion criteria. Ureteral stenosis, anatomical anomalies, ectopic kidney, solitary kidney, and previously inserted ureteral stents were excluded from consideration. The patients were consecutively assigned to NTBS and SAS groups. When sufficient sample size was obtained as a result of the power analysis (a total of 88 patients, NTBS=43, SAS=45), the study was terminated, and the patients’ data were analyzed in a retrospective manner.

Non-contrast computerized tomography (NCCT) and kidney-ureter-bladder graphy were employed in the pre-treatment radiological evaluation procedures to evaluate the lower calyceal anatomy and any relevant stone-related parameters. Urinary ultrasonography was also conducted when needed. In NCCT, the size of the stones was assessed by using the largest diameter of the stone.

A negative urine culture was verified in every case prior to surgery. Patients with documented urinary tract infections were given adequate antibiotic treatment. Just before surgery, all patients received a single dose of second-generation cephalosporin as a prophylactic antibiotic. The NCCT was employed to assess the stone-free status after 1 month following treatment. Cases with either no residual fragment or with fragments smaller than 3 mm were accepted as stone-free.

**Figure 1:**
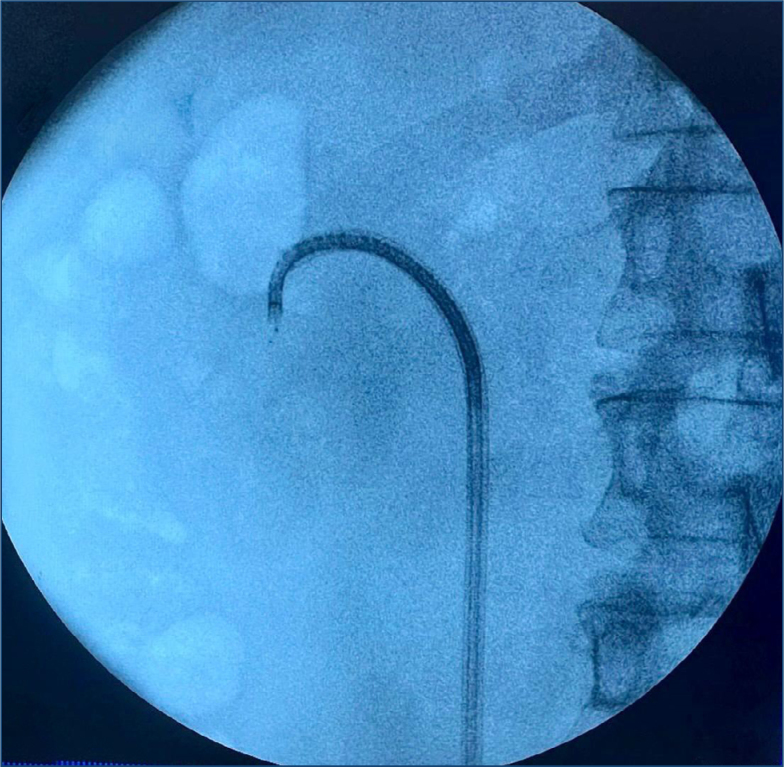
The fluoroscopic view of the novel tip-bendable suction ureteral access sheath, which was pushed to the lower renal calyx target position.

Both the flexible ureterorenoscope (URS) and laser systems used for RIRS were the same brand and model in the two centers where the study protocols were carried out. Additionally, the same accessory equipment, such as the guide wire and access sheaths, was used during the procedures. The surgeries in both centers were carried out by two surgeons with more than 10 years of endourology experience, trained by the same mentor. A general anesthetic and lithotomy position were used for all procedures. Using a 9.5 Fr semi-rigid URS and a 0.038 Fr guide wire, the renal pelvis was reached under fluoroscopic supervision. A pelvicalyceal system assessment was conducted through retrograde pyelography. Group SAS inserted a UAS (9.5/11.5 Fr, Cook Medical, Bloomington, IN) under fluoroscopic guidance. A pressure-regulating ventilation slit and an appropriate suction effect determined by the vacuum's suction parameter were made possible by connecting the suction channel to the vacuum device in Group NTBS once it was confirmed that the NTBS (ClearPetra; Well Lead Medical, Guangzhou, China) tip was at the ureteropelvic junction. Following the inspection of the collecting system, the flexible URS was retracted into the end opening of the NTBS. Under the supervision of the flexible URS, the NTBS was pushed to the lower renal calyx target position (Figure 1). A holmium fiber (Litho 30 W Holmium laser, Quanta System, Samarate, Italy) was utilized to fragment stones with a 273-μm fiber. Either way, the stones in the lower pole were not moved and disintegrated in situ. The laser was calibrated at 0.5 J–20 Hz for the lithotripsy.

### Statistical analysis

For the statistical analysis, Statistical Package for the Social Sciences (SPSS) 22.0 was utilized (SPSS Inc., Chicago, IL, USA). The median interquartile range was used for expressing continuous variables. Mann-Whitney U tests and t-tests were used to compare these variables. Count and percentage were used to represent the categorical variables. These variables were compared using Fisher's exact test and chi-square test. To determine the effect size (Cohen's d) and power value (1-β) for stone-free rates between the two patient groups, we utilized the G*Power program (version 3.1.9.2). The alpha level used to conduct this study was 0.05. The power and effect size values were 0.803 and 0.3, consequently.

## RESULTS

A total of 88 patients were included, with Group 1 including 45 cases undergoing RIRS with SAS and Group 2 including 43 cases treated with NTBS. Patient characteristics were similar in both groups. Additionally, there was no significant difference regarding the presence of diabetes mellitus, the incidence of anticoagulant use, the mean value of the body mass index, and the mean score of the Charlson Comorbidity Index between the two groups. Renal functional status (assessed by serum creatinine levels) and the stone-related parameters (size, number, lateralization, and density) were found to be similar. The mean value of infundibulopelvic angle and the degree of hydronephrosis were again in the same range between both groups (Table 1).

**Table 1:** Patients’ characteristics and clinical findings.

		Group NTB (n=43)		Group SAS (n=45)		p
Gender^a^	Male	29	67.4%	27	60%	0.308
	Female	14	32.6%	18	40%	
Age^b^		56	[39–69]	55	[42–61]	0.356
Diabetes mellitus^a^		5	11.6%	3	6.7%	0.331
Body mass index (kg/m²)^b^		27	[23–32]	26	[22–29]	0.214
Charlson Comorbidity Index^b^		1	[1–3]	1	[0–2]	0.223
Glomerular filtration rate (mL/min/1.73 m^2^)^b^		90	[60–120]	95	[64.90–116.90]	0.649
Preoperative creatinine (mg/dL)^b^		0.99	[0.85–1.11]	0.89	[0.77–1]	0.078
Antiagregant/anticoagulant usage^a^		32	74.4%	39	86.7%	0.118
		11	25.6%	6	13.3%	
Stone size (mm)^b^		10	[8–17]	11	[8–13]	0.242
Stone number^a^	Single	31	72.1%	30	66.7%	0.375
	Multiple	12	27.9%	15	33.3%	
Stone laterality^a^	Right	18	41.9%	16	35.6%	0.349
	Left	25	58.1%	29	64.4%	
Stone density (HU)^b^		856	[589–1,017]	884	[642–1,017]	0.548
Opacity^a^	Opaque	34	79.1%	38	84.4%	0.353
	Non-opaque	9	20.9%	7	15.6%	
Infundibulopelvic angle (°)^b^		49	[41–56]	50	[44–57]	0.628
Hydronephrosis^a^		13	30.2%	10	22.2%	0.270

a Data were expressed as the median and interquartile range.

b Data were expressed as count and frequency.

NTB: novel tip-bendable suction ureteral access sheaths; SAS: standard ureteral access sheaths.

Our postoperative data revealed that, although the stone-free rate (SFR) was higher in the NTBS group than in the SAS group, the difference was not statistically significant (81.4 vs. 73.3%, p=0.259). However, evaluation of the mean residual fragment size in both groups demonstrated that the fragments in Group SAS tended to be significantly larger than the ones in the other group (4.25- [3.75–5] vs. 6- [5–7], p=0.01). The mean change in serum creatinine levels compared to the preoperative period did not show a significant difference in both groups. Although the duration of hospital stay did not show a significant difference between the two groups, the operative duration was significantly shorter in favor of Group NTBS (55- [48–65] vs. 62- [59–72], p=0.016). Last but not least, regarding the complications detected in both groups (modified Clavien-Dindo classification), a significantly higher complication rate was detected in Group SAS (9.3 vs. 26.6%, p=0.032) when compared to the other group. The type and incidence of complications along with all postoperative data are presented in Table 2.

**Table 2:** Postoperative follow-up data.

	Group NTB (n=43)		Group SAS (n=45)		p
Stone free rate^b^	35	81.4%	33	73.3%	0.259
Residual fragment size (mm)^a^	4.25	[3.75–5]	6	[5–7]	0.01
Postoperative creatinine (mg/dL)^a^	0.94	[0.84–1.08]	0.91	[0.74–0.98]	0.042
Creatinine change (mg/dL)^a^	0.02	[-0.06 to 0.06]	0.03	[-0.06 to 0.09]	0.285
Hospitalization (day)^a^	2	[2–2]	2	[2–2]	0.290
Operative time (min)^a^	55	[48–65]	62	[59–72]	0.016
Total complications (modified Clavien-Dindo classification)^b^	4	9.3%	12	26.6%	0.032
Postoperative fever^b^	1	2.3%	4	8.9%	0.195
Transient hematuria^b^	3	6.9%	6	13.3%	0.265
Colic due to blood clots^b^	0	0%	1	2.2%	
Subcupsular hematoma^b^	0	0%	1	2.2%	

a Data were expressed as the median and interquartile range.

b Data were expressed as count and frequency.

NTB: novel tip-bendable suction ureteral access sheaths; SAS: standard ureteral access sheaths.

## DISCUSSION

The main finding of our current study was that the mean size of the residual fragments detected after RIRS for lower calyceal stones was significantly smaller in the NTBS group compared to the SAS group. Additionally, this group had a lower rate of total complications and a significantly shorter operation time.

In 2019, Zhu et al. compared the SFR rates of 165 patients undergoing RIRS using suction-assisted UAS and standard UAS during the first postoperative month evaluation and did not see any significant difference between the two groups in this aspect (p=0.13)^9^. In another study conducted by Qian et al. in 162 patients, stone-free rates were found to be similar between suction-assisted UAS and standard UAS during the same postoperative evaluation period (p=0.368)^10^. However, unlike these findings, in their original trial, Zhang et al. found the SFR rate was found to be 91.2% in the NTBS group and 81.3% in the SAS group during the first postoperative month with a significant difference (p=0.037)^11^. But, contrary to our study, the authors included the stones in the renal pelvis and all other calyces. Examination of the SFR in the groups during the first postoperative month did not show any significant difference (p=0.259) in this aspect. On the other hand, the important part of our methodology was to evaluate the size of the residing stone particles after applying both techniques, and no study so far aimed to compare this parameter between these two techniques (NTBS vs. SAS). The only study focusing on this parameter was performed by Chen et al. to compare the post-procedure residual stone volume after applying these two different access sheaths on a pig model in 2022. They concluded that the residual stone volume was significantly smaller in the NTBS group (p=0.017)^12^.

The results of our study showed that cases in Group NTBS had significantly smaller residual fragments than the other group (p=0.01). We assume that this finding may be due to the better view of the surgical field obtained in these cases with the suction feature incorporated. On the contrary, regarding the surgery time between these two groups, Zhang et al. and Zhu et al. were able to show a significantly shorter duration in the NTBS group (p=0.028, p<0.001)^9,11^. Our findings were consistent with prior investigations, with surgical times much shorter in the Group NTBS (p=0.016). It is possible to explain this situation by removing stone dust from the collecting system with the effect of suction, providing a better field of vision, and reducing the fragment load. In the Zhu et al. and Zhang et al. studies, there was no significant difference in hospitalization stay across the groups (p=0.13, p=0.57)^9,11^. In a similar vein, there was no difference in hospital stay between the two groups in our study (p=0.290). When both groups were compared concerning the type and rate of complications, a significantly lower rate was detected in the Group NTBS in our study (9.3 vs. 26.6%, p=0.032). Similar to our findings again, Zhu et al. and Zhang et al., did observe lower complication rates in the suction-assisted groups (11.5 vs. 24.8%, p<0.01; 11.8 vs. 25%, p=0.013), consistent with our study^9,11^. It is conceivable to assume that the suction modality applied in the Group NTBS limited the pressure increase during the procedure, which would have decreased pyelovenous-lymphatic reflux and pyelotubular reflux. It is clear that with this effect, the risk of infection will be lower in these cases. Finally, Erkoc et al. used NTBS (ClearPETRA) and SAS in their study and showed that the use of NTBS during the procedure can reduce the possibility of postoperative fever. They also achieved higher SFR within significantly lower operative time and lower sepsis rates in the NTBS group in the management of stones larger than 2 cm^13^. The total complication rate was lower in favor of Group NTBS in our study, despite the fact that we did not find a significant difference in the postoperative fever rates in either group. Additionally, the operation time was significantly shorter in this group.

### Limitations

There are certain limitations of the present research. The first potential drawback would be the small number of patients that were included. Moreover, the study did not use a double-blind, randomized approach, even though the data were noted and collected prospectively. A retrospective analysis of the data was conducted. We think that our results might be sufficient in this regard, considering the scant data that have been published to date.

## CONCLUSION

Endourologists face a unique challenge in terms of the treatment of lower pole stones during RIRS. The findings of this study showed that when treating lower calyceal stones smaller than 2 cm with RIRS, using a new type of bendable access sheath could result in lower residual stone sizes and shorter surgery time. It is evident that more extensive patient series and controlled randomized trials are required for this topic.

## Data Availability

It is not open to data sharing due to personal data.
